# Resilience and associated factors within the mental health profile of incarcerated adults in Portugal: a cross-sectional study

**DOI:** 10.1186/s12888-026-07852-1

**Published:** 2026-01-29

**Authors:** Mariana Alfaiate, Lino Ramos, Ana Morais, Francisco Sampaio

**Affiliations:** 1 National School of Public Health, ENSP, Public Health Research Centre, Comprehensive Health Research Center, CHRC, REAL, CCAL, NOVA University Lisbon, Lisbon, Portugal; 2 School of Health and Human Development, Universidade de Évora, Évora, Portugal; 3 NOVA, Medical School, University Lisbon, Lisbon, Portugal; 4 RISE-Health, Nursing School of Porto, Porto, Portugal; 5 Directorate General of Reintegration and Prison Services - Leiria Prison Establishment, Leiria, Portugal; 6 Comprehensive Health Research Centre (CHRC), University of Évora, Évora, Portugal; 7 School of Health, Polytechnic Institute of Setúbal, Setúbal, Portugal; 8 Nursing School of Porto, Porto, Portugal

**Keywords:** Mental health, Prisons, Incarceration, Resilience, Adults

## Abstract

**Background:**

Mental disorders are widely recognised as a major contributor to morbidity in prisons across the European Union, with evidence consistently indicating a markedly higher mental health burden among incarcerated individuals compared with community populations. Prison research has mainly focused on mental illness, often neglecting protective aspects of mental health from a salutogenic perspective. In Portugal, previous studies have examined specific mental health outcomes in prison populations, such as suicidal behaviour, personality pathology, trauma-related symptoms, or substance use; however, integrative research simultaneously encompassing multiple mental health symptoms, positive psychological constructs, and associated psychosocial factors remains limited. This study seeks to address this gap by evaluating mental health symptoms (stress, anxiety and depression), substance use, and positive psychological constructs (resilience, hope and psychological well-being) among adult incarcerated individuals and to explore factors associated with resilience as a secondary, hypothesis-generating analysis.

**Methods:**

A cross-sectional study was conducted with 576 incarcerated individuals in mainland Portugal. Data were collected using the 10-item Connor–Davidson Resilience Scale (CD-RISC-10), the 21-item Depression, Anxiety and Stress Scale (DASS-21), the Herth Hope Index (HHI), the Psychological Well-Being Manifestation Scale (PWBM-S), five items from the Alcohol, Smoking and Substance Involvement Screening Test (ASSIST), and a sociodemographic and legal-criminal questionnaire. Multiple mental health domains were characterised and explored factors associated with resilience as a secondary hypothesis-generating analysis. Multivariable linear regression was used to examine associations between resilience and candidate sociodemographic, clinical, and psychosocial factors.

**Results:**

The DASS-21 indicated mean scores of 4.7 for anxiety, 4.9 for depression, and 6.2 for stress. Mean scores on the CD-RISC-10 29.1, HHI 31.2, and PWBM-S 96.9 are reported. The greatest risks of substance dependence were observed for tobacco, alcohol, cannabinoids and cocaine. Multiple linear regression suggested psychological support prior to incarceration, an open prison regime, the ability to cope with negative emotions, engagement in physical activity, face-to-face contact with family and friends, practice of relaxation techniques and regular reflection on the reasons for incarceration were positively associated with higher resilience levels. By contrast, exposure to verbal and/or physical aggression was inversely associated with resilience levels.

**Conclusions:**

These findings highlight the urgent need to design and implement structured, multidimensional, and evidence-based interventions to strengthen resilience and improve mental health among people in prison.

**Clinical trial number:**

Not applicable.

**Supplementary Information:**

The online version contains supplementary material available at 10.1186/s12888-026-07852-1.

## Background

### Mental health burden and adaptation in custodial settings

Mental health in prisons was highlighted in the World Health Organization’s 2023 report on the European Union, which identified mental disorders as the leading cause of morbidity in European prisons, with an estimated prevalence of 32.8%, compared with 13.1% in the general population [1]. Adaptation to the prison environment varies considerably between individuals and should not be regarded as a uniform or impersonal process. Emotional disturbances and difficulties in adjustment are commonly observed [2]. This adaptation may be conceptualised as an acculturation process, whereby individuals internalise the rules, routines, and behavioural expectations imposed by the prison context. Incarceration involves not only compliance with institutional norms but also the assimilation of characteristic ways of thinking, feeling, and behaving that align with the prison culture [3]. Such processes help explain the markedly higher prevalence of mental disorders among incarcerated populations compared with the general population [4]. In recent years, mental health has gained increasing prominence on political agendas, particularly with regard to vulnerable groups such as those in prison [5].

### Mental health morbidity in prison populations

Recent research highlights a high prevalence of psychopathology in prison populations, including anxiety, stress, depression, psychosis, suicidal ideation, substance use disorders, and personality disorders [6, 7]. These conditions occur irrespective of age, gender, or type of correctional facility [8, 9].

Mental health in prison should be understood as a dynamic and multifactorial phenomenon, shaped by complex and non-linear interactions between individual vulnerabilities, prior life experiences, and institutional conditions. Rather than reflecting simple cause–effect relationships, mental health outcomes in prison are examined in the present study in terms of correlates or associated factors, and no assumptions regarding directionality or causality can be inferred. The observed relationships should therefore be interpreted as complex, context-dependent, and potentially bidirectional, particularly with regard to mood- and anxiety-related symptoms, whose aetiology is widely recognised as multifactorial [10].

### Factors associated with mental health in prison

A range of factors are associated with the mental health of incarcerated individuals, encompassing both risk and protective dimensions that operate across individual, relational, and institutional levels. These factors should not be interpreted as unidirectional or causal, but rather as interconnected elements within a broader psychosocial system. Negatively associated factors include isolation from family and peers, limited opportunities for social engagement, and adverse relational dynamics between staff and prisoners [10, 11]. Conversely, older age, engagement in physical activity, emotional regulation capacities, and resilience have consistently been identified as protective correlates of mental health in prison settings [10–12].

Family visits play a pivotal role in maintaining and strengthening social bonds, serving as protective factors for both mental health and general well-being [13, 14]. Such visits provide meaningful relief from the monotony of prison life, foster emotional support, and facilitate continued connection with external reality. They also reinforce familial roles, particularly in reaffirming parental identity for those with children [15].

The prison environment—characterised by restricted freedom, social isolation, institutional rigidity, and often interpersonal violence—demands robust emotional regulation for effective adaptation.

### Psychological adaptation and protective processes in prison: emotion regulation, resilience, and hope

Emotion regulation, particularly the capacity to process and manage negative emotions such as anger, fear, and sadness, plays a central role in psychological adjustment to incarceration. Rather than acting as a single determinant, emotion regulation interacts with environmental stressors and personal resources, influencing vulnerability or resilience to psychological distress. Empirical evidence suggests that individuals who employ more adaptive emotion regulation strategies report better mental health outcomes, including lower levels of anxiety and depression, greater resilience, and fewer disciplinary infractions [15–17]. Resilience has been defined as a set of phenomena characterised by positive outcomes despite serious threats to adaptation or development [18], or as personal qualities that enable an individual to thrive in the face of adversity [19]. In the prison setting, resilience constitutes a key protective factor, enabling individuals to cope more effectively with the emotional and structural demands of incarceration, particularly in contexts characterised by limited social support. It mitigates psychiatric morbidity and fosters psychological stability [20]. Evidence indicates that levels of mental health, resilience, self-acceptance, and perceived social support among prisoners are generally low and significantly shaped by sociodemographic variables. Nonetheless, self-acceptance, perceived social support, and resilience function as protective factors for mental health, with self-acceptance and resilience mediating the association between social support and psychological outcomes [21]. Consistently, greater resilience is linked to lower levels of depression, anxiety, and self-harming behaviours, alongside improved emotional adjustment and a stronger capacity to manage the deprivation of liberty [19, 21, 22]. Hope similarly constitutes a crucial component of psychological adjustment in prison, being associated with lower levels of distress, more effective coping strategies, and stronger engagement with rehabilitative programmes [23–25]. With respect to psychological well-being, studies indicate that prisoners with lower levels of well-being are more likely to experience mental health problems throughout their sentence [26]. It is equally important to assess symptoms of depression, anxiety, and stress, given their high prevalence in prison populations, with rates consistently exceeding those observed in the general population. Substance use is another major concern, as substance use disorders are disproportionately common among prisoners [6, 7].

### Mental health in Portuguese prisons and study rationale

International evidence consistently demonstrates that mental health in prisons is not merely an individual concern but also a pressing public health issue, as prisoners are recognised as a particularly vulnerable group, presenting disproportionately high rates of mental disorders. In Portugal, however, there is a lack of national data characterising the mental health of the prison population [1]. Empirical research conducted in Portuguese prison settings has examined specific mental health outcomes among incarcerated men, including suicidal behaviours, personality disorders, trauma-related symptoms, and substance use, often within single institutions or local samples. While these studies provide important contributions to understanding particular dimensions of prison mental health, they have typically focused on isolated domains rather than adopting a multidimensional approach that simultaneously integrates negative mental health symptoms, positive psychological constructs, and associated psychosocial factors [27–30].

Within the European prison mental health literature, many studies have prioritised the description of psychiatric morbidity or the examination of individual-level correlates. Although multicentre and national studies do exist, approaches that simultaneously integrate mental health symptoms, substance use, and positive psychological resources, while also considering contextual prison-level factors, remain comparatively less common. The present study contributes to this field by adopting a multidimensional assessment of mental health and psychological adaptation among incarcerated adults, integrating symptoms, positive psychological constructs, and associated factors within a national sample in Portugal.

In the absence of national studies examining mental health symptoms, substance use, and psychosocial resources among adults in prison in Portugal, a multidimensional assessment is warranted. Such an assessment should include resilience, hope, psychological well-being, and symptoms of depression, anxiety, and stress, as well as substance use, since these domains are closely related to adaptation, emotion regulation in prison, and successful social reintegration after release [4, 31, 32].

The present study does not seek to provide a comprehensive psychiatric diagnostic profile of incarcerated adults. Rather, it adopts a multidimensional mental health assessment approach encompassing mental health symptoms (stress, anxiety, and depression), substance use, and positive psychological constructs (resilience, hope, and psychological well-being), selected for their relevance to psychological adaptation and functioning in custodial settings.

Accordingly, the primary aim was to describe multiple mental health domains in incarcerated adults. Following the descriptive profile, we conducted an exploratory analysis of factors associated with resilience, given its clinical relevance as a potentially modifiable protective factor.

## Methods

### Study setting: Portuguese prison system

The Portuguese prison system encompasses different regimes of sentence execution. The common regime represents the most restrictive model, characterised by high levels of surveillance, severe limitations on mobility, and fewer opportunities for activities. The security regime is applied to prisoners considered to pose higher levels of risk or dangerousness, involving increased control and additional restrictions. The open regime within prison provides greater autonomy to prisoners inside the facility, emphasising individual responsibility and participation in labour, educational, or recreational activities. The open regime outside prison is the least restrictive, allowing engagement in activities in the community and intended for low-risk prisoners, functioning as preparation for social reintegration and eventual conditional release [33].

### Study design and population

The cross-sectional study was conducted between February and March 2025. The target population comprised individuals incarcerated in prisons across mainland Portugal. A non-probability convenience sampling method was employed for participant recruitment.

The study was conducted across 14 prison establishments in mainland Portugal, distributed across the Northern (*n* = 2), Central (*n* = 6), and Southern (*n* = 6) regions, according to the official territorial organisation defined by the jurisdictions of the Courts for the Execution of Sentences. The selected establishments reflect institutional diversity in terms of prison regime, security level, and population profile (e.g., remand versus sentenced populations). For data protection and confidentiality reasons, and in accordance with requirements from the Data Protection Officer, individual prison establishments are not identified.

Whilst the national prison population was approximately 12,000 individuals, an initial sample size estimate was obtained using the sample size calculator available in SurveyMonkey, assuming a 95% confidence level and a 5% margin of error. This estimate was used solely as a pragmatic reference to guide recruitment. Given the exploratory nature of the study, the inclusion of multiple outcomes, and the use of a non-probability convenience sampling strategy, no a priori hypothesis-driven power analysis based on an assumed effect size or specific statistical tests was conducted.

To address the adequacy of the sample size for the main multivariable analysis, a sensitivity analysis was performed using GPower (F test; linear multiple regression, fixed model, R² deviation from zero; α = 0.05; power = 0.80). Assuming up to 18 predictors/parameters in the multivariable model, the minimum detectable effect size was Cohen’s f² ≈ 0.036, corresponding to R² ≈ 0.035. A final sample of 576 participants was achieved, providing a robust basis for descriptive and exploratory analyses within the study sample; however, the findings should be interpreted as descriptive rather than statistically representative of the Portuguese prison population.

The inclusion criterion was: (a) incarcerated individuals aged 18 years or older. The exclusion criteria were: (a) inability to communicate (read and/or write) in Portuguese; and (b) a prior clinical diagnosis of a neurodevelopmental disorder (including mild, moderate, or severe intellectual disability; speech and/or language disorder; or level 2 or 3 autism spectrum disorder) and/or a neurocognitive disorder (such as Alzheimer’s disease, frontotemporal degeneration, Lewy body disease, prion disease, or Huntington’s disease).

Eligible incarcerated individuals were initially invited to participate in the study. Those who agreed were subsequently scheduled to attend a data collection session. Questionnaires were administered in designated rooms accommodating small groups of approximately six to eight participants, with each individual seated at a separate desk to allow for independent and confidential completion. All participants who consented and attended the scheduled data collection session completed the questionnaires in full. No dropouts occurred between consent and questionnaire administration.

The sample consisted exclusively of a male prison population. The decision to focus the study solely on men was based on the fact that they represent the overwhelming majority of individuals deprived of liberty in Portugal (over 90%). Furthermore, the significantly smaller number of female prison facilities would have hindered the recruitment of a sufficiently large female sample, potentially compromising the robustness and validity of the results.

### Data collection and measures

With regard to the instruments employed, a structured Sociodemographic and Legal-Criminal Questionnaire was developed to support sample characterisation and the collection of contextual variables relevant to mental health in prison settings, informed by previous empirical literature (see Supplementary File 1). The questionnaire comprised 19 items organised into four domains and was not designed as a psychometric instrument, as it does not measure latent constructs nor generate composite scores. The first domain encompassed personal information, including age, parental status, and history of psychological support or prior diagnosis of a mental disorder. The second domain addressed issues related to the type of prison regime and the duration of incarceration to date. The third domain included opinion-based items concerning the prison context, such as the availability of stimulating activities, the ability to manage negative emotions, the existence of adequate release planning, and perceived stigma associated with imprisonment. These items were included as exploratory contextual indicators and were analysed individually. The fourth domain incorporated variables related to social contact with family and friends (in person, by telephone, or by letter), participation in physical, religious, and relaxation activities, exposure to verbal or physical aggression, and the frequency of thoughts regarding the reason for incarceration.

The risks or problems associated with the use of tobacco, alcohol, cannabis, cocaine, amphetamine-type stimulants, inhalants, sedatives, hallucinogens, opioids, and other substances were assessed using a reduced set of five questions adapted from the Alcohol, Smoking and Substance Involvement Screening Test (ASSIST) [34, 35]. Item selection was guided by ethical and contextual considerations specific to the prison setting, including the sensitivity of substance use disclosure, the need to minimise respondent burden and social desirability bias, and the intention to reduce item non-response and potential sample loss. The selected items were analysed individually to capture substance use across key categories rather than to generate a composite risk score; therefore, internal consistency metrics such as Cronbach’s alpha were not calculated.

The first, third, and fourth questions were scored on a scale ranging from 0 to 3 points, while the second question was scored on a scale of 0, 3, 4, 5, and 6 points, following the original ASSIST scoring logic. Higher scores indicated a greater level of substance-related risk. The final question identified the proportion of the sample that had used injectable substances without medical prescription (Q1: Which of the following substances have you ever used throughout your life?; Q2: Over the past three months, how often have you felt a strong craving or urge to use any substance?; Q3: Has a friend, relative, or anyone else ever expressed concern about your use of any of the following substances?; Q4: Have you ever tried, without success, to reduce or stop your use of any of the following substances? Q5: Have you ever used injectable substances without a medical prescription? Higher scores indicated a greater level of substance-related risk. Scores were calculated for descriptive purposes only, in order to compute mean values and to explore patterns of risk of dependence across different substances.

Depression, anxiety, and stress were assessed with the Portuguese version of the Depression, Anxiety and Stress Scale – 21 (DASS-21) [36, 37]. This scale consists of 21 items, with seven items for each domain—depression, anxiety, and stress—and offers four response options. The DASS is a dimensional measure of symptom severity, rather than a diagnostic tool, and should therefore be treated as a continuous variable [36]. The Portuguese version demonstrated good psychometric properties, with satisfactory internal consistency coefficients (Cronbach’s α) for depression (α = 0.85), anxiety (α = 0.74), and stress (α = 0.81) [37]. In the present study, with the scale presented reliability coefficients of 0.86 for depression, 0.88 for stress, and 0.87 for anxiety.

Resilience was measured using the 10-item Connor–Davidson Resilience Scale (CD-RISC-10), a self-report questionnaire designed to assess the individual’s capacity to cope with stress and adversity. The CD-RISC-10 comprises 10 items rated on a 4-point Likert scale, ranging from “not true at all” to “true nearly all the time” [18, 38]. Higher scores indicate greater resilience, whereas lower scores indicate reduced resilience or greater difficulty in recovering from adversity. As no validated clinical cut-off points exist for this version, resilience was treated as a continuous construct. In the Portuguese validation study, the CD-RISC-10 demonstrated good internal consistency (Cronbach’s α = 0.84) [39]. In the present study, internal consistency was also strong (Cronbach’s α = 0.88).

Hope was assessed using the Portuguese version of the Herth Hope Index (HHI-PT-9), consisting of nine items rated on a 4-point Likert scale ranging from “strongly disagree” to “strongly agree” [40] As no clinically established cut-off scores exist, hope was treated as a continuous variable, with higher scores reflecting greater perceived hope [41]. In the Portuguese cultural adaptation, the HHI-PT-9 demonstrated high internal consistency (Cronbach’s α = 0.873) [42]. In the present study, the scale demonstrated excellent reliability (Cronbach’s α = 0.89).

Psychological well-being was assessed using the Psychological Well-Being Manifestation Scale (PWBM-S), which comprises 25 items rated on a 5-point Likert scale ranging from “never” to “almost always” [43]. Higher scores indicate greater psychological well-being. In the Portuguese validation study, the PWBM-S demonstrated excellent internal consistency (Cronbach’s α ≈ 0.92) [44]. In the present study, the internal consistency of the full 25-item scale was also high (Cronbach’s α = 0.93).

Data collection was conducted in person by the lead investigator across 14 prisons in mainland Portugal, using paper-based questionnaires and in full compliance with each institution’s safety protocols and regulations. The selection of prison establishments was informed by several factors: establishment typology (i.e., predominance of remand versus sentenced populations), security levels, operational complexity, and formal authorisation from the Directorate-General for Reintegration and Prison Services.

Questionnaires were self-administered in small groups of 6–8 participants at a time, in rooms provided by the prison health services that ensured privacy and the absence of prison officers or administrative staff. Prior to participation, all individuals received oral and written information regarding the voluntary nature of the study, the absence of any consequences for declining participation, and the confidentiality and anonymity of responses. Sampling was guided by predefined inclusion and exclusion criteria, as well as participants’ availability and willingness to take part. In view of time and resource constraints, and acknowledging operational limitations within prison settings, each establishment was contacted in advance and clinical services staff compiled a list of potentially eligible individuals, who were then invited to participate by the research team. At no stage did prison management interfere with recruitment or questionnaire administration. Upon completion, questionnaires were deposited by participants into multiple sealed envelopes, in random order, to prevent any possibility of individual identification. Because the total number of individuals approached, eligible, or declining participation could not be reliably quantified across establishments, a formal response rate could not be calculated.

### Statistical analysis

Statistical analyses were performed using IBM SPSS Statistics (version 29). Descriptive statistics were first computed to characterise the sample, including measures of central tendency (mean) and dispersion (standard deviation, minimum, and maximum values). Absolute and relative frequencies were also calculated for categorical variables, providing a comprehensive overview of the participants’ sociodemographic and legal-criminal characteristics.

The distribution of continuous variables was examined for descriptive purposes using the Kolmogorov–Smirnov test, skewness and kurtosis coefficients, and visual inspection of histograms and Q–Q plots. These procedures were used to characterise the data and support descriptive reporting, rather than to determine the choice of regression models.

The internal consistency of the psychometric instruments was evaluated using Cronbach’s alpha coefficient, calculated for each scale and subscale. Values of 0.70 or higher were considered indicative of acceptable internal consistency [45].

In the absence of validated clinical cut-off points for resilience, hope, and psychological well-being, these constructs were treated as continuous variables.

Given the exploratory nature and multiple measured domains, we did not plan to model all outcomes. The descriptive analyses covered all mental health domains assessed. Based on the descriptive profile, resilience was selected for additional exploratory multivariable modelling to identify factors associated with resilience. These analyses were intended to be hypothesis-generating rather than confirmatory.

The primary multivariable model (Model A) was theory-driven and included all covariates defined a priori, encompassing sociodemographic characteristics, legal and prison-context variables, and relevant baseline clinical factors. Key covariates were forced into the multivariable models regardless of statistical significance, in line with the theory-driven analytical approach. This model addressed the question of which contextual factors were associated with resilience among incarcerated adults.

To assess the robustness of the findings after adjustment for concomitant psychological symptomatology, sensitivity analyses (Models B) were conducted using the Depression, Anxiety and Stress Scale (DASS-21) (see Supplementary File 2). In a first, exploratory step, the stress, anxiety and depression subscales were entered simultaneously into the multivariable model to examine their joint behaviour in relation to resilience. Given the instability associated with intercorrelations among the subscales, three additional (primary) sensitivity models were subsequently estimated, in which stress, anxiety and depression were entered separately (see Supplementary File 3, 4 and 5).

After estimating Model A and the sensitivity analyses (Models B), variables were compared across models to identify those that remained stable. Variables were considered stable when they were retained in Model A and in all Models B, with a consistent direction of association with resilience.

Penalised regression analyses (Elastic Net/LASSO) were subsequently conducted as an additional robustness assessment. In the Elastic Net analysis, given that the penalised model retained a large number of variables, results were synthesised descriptively, reporting only the predictors with the largest absolute values of the standardised coefficients (|β|), as well as the corresponding category (dummy) in which this maximum value was observed (see Supplementary File 6). The LASSO procedure was used to further assess the stability of variable selection by imposing stronger coefficient shrinkage, thereby identifying predictors that remained retained under more stringent penalisation (see Supplementary File 7).

Normality of residuals was assessed through visual inspection of residual histograms and normal probability (P–P) plots. Linearity and homoscedasticity were examined using plots of standardised residuals versus standardised predicted values. Multicollinearity was assessed using variance inflation factors (VIF) and tolerance statistics. Model fit was evaluated using R², adjusted R², and the overall F-test. No substantial violations of regression assumptions were identified that required data transformation or alternative modelling approaches.

Formal mediation or moderation analyses were not undertaken, as the study was designed to examine associations rather than mechanistic pathways, and the cross-sectional design precludes causal inference.

### Ethical considerations

Following approval from the Ethics Committee and the Data Protection Officer of the University of Évora (Approval No. 24055), authorisation to conduct the study was formally requested through an application submitted to the Director of the Directorate-General for Reintegration and Prison Services.

## Results

### Legal-criminal and sociodemographic questionnaire and mental health characterization

The sample comprised 576 incarcerated individuals, all of male sex. Regarding parental status, 60.8% reported having children. Prior to incarceration, 33.0% of participants reported having received psychiatric or psychological care. Among these, substance use (27.4%) and depression (14.2%) were the most frequently reported reasons, while 13.7% reported not knowing the reason for previous mental health care. A prior diagnosis of a mental disorder was reported by 13.9% of participants. Among those reporting a diagnosis, schizophrenia (23.8%), personality disorders (17.5%), substance use disorders (15.0%), and depression were reported. At the time of data collection, 14.1% of participants reported a current diagnosis of a mental disorder, most frequently schizophrenia (34.6%), personality disorders and depression (17.3% each), and substance use disorders (11.1%).

With respect to prison regime, 78.2% of participants were serving under the common regime, 11.1% under the open regime within prison, 8.3% under the security regime, and 2.3% under the open regime outside prison.

Regarding the availability of stimulating and diverse activities for personal, emotional, and professional development within the prison context, 44.8% of participants reported agreement or strong agreement with the statement, 38.0% reported disagreement, and 17.7% reported a neutral position.

Concerning the ability to cope with negative emotions, 65.1% reported agreement or strong agreement, 17.7% reported disagreement, and 17.2% reported a neutral position.

Regarding the existence of reintegration planning tailored to individual needs, 43.1% of participants reported agreement or strong agreement, 43.1% reported disagreement, and 13.9% reported a neutral position.

With respect to perceptions of prejudice associated with imprisonment, 57.1% reported agreement, 24.1% reported disagreement, and 18.8% reported a neutral position.

In relation to face-to-face contact with family and friends, 28.8% reported contact two or more times per week, 20.1% once per week, 23.6% monthly contact, and 9.7% fortnightly contact, while 17.7% reported never having face-to-face contact.

Regarding contact by letter or telephone, 66.0% reported contact more than twice per week, whereas 9.2% reported no contact.

With respect to physical activity, 34.0% of participants reported exercising four or more times per week, 26.4% reported never engaging in physical activity, and the remaining participants reported frequencies ranging from once to three times per week.

Regarding the practice of relaxation techniques, 68.6% reported never practising such activities, 15.8% reported practising once per week, 5.4% twice per week, 3.1% three times per week, and 7.1% four or more times per week.

In relation to experiences of verbal and/or physical aggression within the prison context, 64.2% reported never having experienced aggression, 14.9% reported experiencing aggression once per month, 7.3% twice per month, 4.5% three times per month, and 9.0% four or more times per month.

In relation to religious practices, such as praying, attending mass in person or via radio/television, and participating in support groups, 45.0% of participants reported not engaging in any religious activity. In contrast, 22.2% reported weekly participation. Lower frequencies were also reported, including fortnightly practices (12.2%), twice per week (4.5%), and more than twice per week (16.1%).

Regarding the frequency with which incarcerated individuals reflected on or revisited the reasons for their incarceration, the majority (72.6%) reported doing so more than twice a week, whereas 8.5% indicated that they never engaged in such reflection.

The sociodemographic and incarceration characteristics of the incarcerated adults included in the study are presented in Table 1.

**Table 1:** Sociodemographic and incarceration characteristics of the sample

Variables	*n*	(%)	Mean	SD	Max	Min
Age			39.2	12.0	78	18
Time spent in incarceration (days)			879.2	903.0	6450	4

Table 2 presents descriptive statistics for mental health outcomes, while Table 3 summarises descriptive statistics related to psychoactive substance use.

**Table 2:** Descriptive statistics of mental health outcomes among incarcerated adults

Variables	Mean	SD	Min	Max
**DASS − 21**				
Subscale anxiety	4.7	5	0	21
Subscale depression	4.9	5	0	21
Subscale stress	6.2	5.2	0	21
**CD RISC-10**	29.1	7.3	3	40
**HHI-PT**	31.2	4.4	9	36
**PWBM-S**	96.8	17.0	36	125

**Table 3:** Descriptive statistics of psychoactive substance use

Psychoactive substance	*n*	(%)	Mean	SD	Max	Min	Total score (Σ)
Tobacco	462	80.2	11.6	3.6	15	3	5357
Alcoholic drinks	450	78.1	6.9	3.5	15	3	3099
Cannabinoids	324	56.3	8.1	3.9	15	3	2611
Cocaine	200	34.7	8	3.7	15	3	1598
Stimulants	133	23.1	6.7	3.4	15	3	892
Inhalants	32	5.6	5.6	3.5	15	3	180
Anxiolytics	87	10.1	7.8	4.3	15	3	677
Hallucinogens	92	16	6.6	3.7	15	3	606
Opiates	118	20.5	8	3.9	15	3	941
Other psychoactive substances	8	1.4	5.3	2.9	11	3	42
Use of injection drugs	63	10.9					

### Multiple linear regression analysis of factors associated with resilience

Multivariable linear regression analysis was conducted to examine factors associated with resilience among incarcerated adults. The primary analysis was based on a theory-driven multivariable model (Model A), in which all predefined sociodemographic, legal–criminal, clinical history, and prison-context variables were entered simultaneously, irrespective of univariable statistical significance.

In Model A, the coefficient of determination was R² = 0.249, with an adjusted R² = 0.172. The standard error of the estimate was 6.629, and the Durbin–Watson statistic was 1.890, indicating no evidence of substantial residual autocorrelation. These indices describe the proportion of variance in resilience accounted for by the set of variables included in the model.

After simultaneous adjustment for all predefined covariates, the following variables were retained in the model and showed positive associations with resilience: prior psychological or psychiatric support before incarceration; placement in an open prison regime within the facility; higher self-reported ability to manage negative emotions; frequent engagement in physical activity; regular face-to-face contact with family and friends; frequent practice of relaxation techniques; and regular reflection on the reasons for incarceration. Exposure to verbal and/or physical aggression within the prison context was retained in the model with a negative association with resilience.

The results of the primary theory-driven multivariable linear regression model (Model A), in which all predefined covariates were entered simultaneously, are presented in Table 4.

**Table 4:** Multiple linear regression analysis of factors associated with resilience

Variable	Unstandardized B	*P*-Value	95% CI for B
Age	0.039	*p* = 0.163	[-0.016; 0.093]
Has children (Yes)	-0.082	*p* = 0.903	[-1.394; 1.230]
**Psychological support before incarceration (Yes)**	**1.611**	*p* = 0.022	**[0.235; 2.987]**
History of diagnosed mental disorder prior to incarceration (yes)	1.103	*p* = 0.360	[-1.261; 3.467]
Current diagnosed mental disorder (yes)	0.004	*p* = 0.997	[-2.232; 2.240]
Time incarcerated (days)	-4.272E-5	*p* = 0.898	[-0.001; 0.001]
Prison regime			
The common prison regime	Ref		
The security regime	1.927	*p* = 0.084	[-0.261; 4.116]
** The open regime within prison**	**2.489**	*p* = 0.010	**[0.591; 4.386]**
The open regime outside prison	1.331	*p* = 0.503	[-2.569; 5.232]
Stimulating activities			
I completely disagree	Ref		
I disagree	-0.052	*p* = 0.961	[-2.150; 2.046]
I neither agree nor disagree	− 0.0593	*p* = 0.573	[-2.660; 1.473]
I agree	-0.537	*p* = 0.588	[-2.484; 1.410]
I completely agree	-0.582	*p* = 0.601	[-2.771; 1.606]
The ability to cope with negative emotions			
I completely disagree	Ref		
I disagree	-1.551	*p* = 0.298	[-4.476; 1.374]
I neither agree nor disagree	0.294	*p* = 0.828	[-2.363; 2.950]
I agree	2.166	*p* = 0.086	[-0.307; 4.640]
** I completely agree**	**4.259**	*p* = 0.002	**[1.580; 6.937]**
There is adequate planning for reintegration			
I completely disagree	Ref		
I disagree	-1.464	*p* = 0.139	[-3.406; 0.478]
I neither agree nor disagree	-1.076	*p* = 0.324	[-3.215; 1.064]
I agree	-0.045	*p* = 0.962	[-1.917; 1.826]
I completely agree	-0.757	*p* = 0.500	[-2.960; 1.446]
There is prejudice due to having been incarcerated			
I completely disagree	Ref		
I disagree	-0.276	*p* = 0.825	[-2.733; 2.180]
I neither agree nor disagree	-1.106	*p* = 0.374	[-3.548; 1.336]
I agree	-0.406	*p* = 0.726	[-2.680; 1.868]
I completely agree	-0.110	*p* = 0.924	[-2.367; 2.148]
Face-to-face contact with family and friends			
Never	Ref		
** Once a month**	**2.022**	*p* = 0.028	**[0.217; 3.827]**
** Once every two weeks**	**3.922**	*p* = 0.001	**[1.592; 6.253]**
** Once a week**	**3.478**	*p* < 0.001	**[1.534; 5.423]**
** Twice or more times a week**	**2.607**	*p* = 0.007	**[0.703; 4.512]**
Contact by letter or telephone with friends or family			
Never	Ref		
Once every two weeks	1.768	*p* = 0.198	[-0.927; 4.463]
Once a week	-0.839	*p* = 0.539	[-3.520; 1.842]
Twice a week	-1.246	*p* = 0.412	[-4.230; 1.737]
More than twice a week	-0.067	*p* = 0.950	[-2.146; 2.012]
Physical activity			
Never	Ref		
Once a week	1.422	*p* = 0.120	[-0.372; 3.215]
Twice a week	0.002	*p* = 0.998	[-2.020; 2.025]
Three times a week	1.811	*p* = 0.092	[-0.298; 3.920]
** Four or more times a week**	**2.845**	*p* < 0.001	**[1.173; 4.517]**
Practice of relaxation techniques			
Never	Ref		
Once a week	-0.839	*p* = 0.312	[-2.469; 0.791]
Twice a week	1.176	*p* = 0.371	[-1.406; 3.758]
Three times a week	0.071	*p* = 0.967	[-3.285; 3.426]
** Four or more times a week**	**2.466**	*p* = 0.039	**[0.127; 4.806]**
Experiences of verbal and/or physical aggression			
Never	Ref		
** Once a month**	**-1.788**	*p* = 0.039	**[-3.489; -0.087]**
Twice a month	-1.010	*p* = 0.382	[-3.276; 1.257]
Three times a month	0.219	*p* = 0.878	[-2.577; 3.015]
Four or more times a month	-1.420	*p* = 0.185	[-3.521; 0.680]
Religious practices			
Never	Ref		
Once every two weeks	0.142	*p* = 0.881	[-1.716; 2.000]
Once a week	1.101	*p* = 0.153	[-0.410; 2.613]
Twice a week	-1.647	*p* = 0.258	[-4.503; 1.209]
More than twice a week	0.947	*p* = 0.277	[-0.762; 2.656]
Reflect on or revisit the reasons for their incarceration			
Never	Ref		
Once every two weeks	2.806	*p* = 0.061	[-0.126; 5.738]
** Once a week**	**3.596**	*p* = 0.015	**[0.711; 6.481]**
Twice a week	-1.037	*p* = 0.520	[-4.201; 2.126]
More than twice a week	0.918	*p* = 0.385	[-1.158; 2.994]

Note: The bold values indicate significance at *p* < 0.05

From the robustness analysis comparing Model A and Models B, the following variables remained stable across all models: open regime within the prison setting, regular face-to-face contact with family and friends (≥ once every two weeks), and frequent engagement in physical activity (≥ four times per week), all of which were associated with higher resilience. Reflecting on the reasons for incarceration once per week (vs. never) also maintained a consistent association. Prior psychological support was attenuated and no longer reached statistical significance in Models B, remaining close to the threshold after adjustment for stress. The ability to cope with negative emotions and the use of relaxation techniques did not constitute stable predictors. Exposure to verbal and/or physical aggression showed a negative association in some models but was not robust across all adjustments.

From the penalised regression analyses (Elastic Net/LASSO), the Elastic Net model indicated that frequent engagement in physical activity exhibited the largest absolute |β| value in the model (|β| = 0.464), corresponding to the category [Four or more times a week = 1]. This was followed by psychological support prior to incarceration (|β| = 0.335; [Psychological support before incarceration = 1]), the ability to cope with negative emotions (|β| = 0.331; [I completely agree = 1]), regular face-to-face contact with family and friends (|β| = 0.313; [Once every two weeks = 1]), a more open prison regime (RAI; |β| = 0.200; [The open regime within prison = 1]), and age (|β| = 0.179).

In the LASSO analysis, only a highly parsimonious subset of variables was retained, predominantly indicators related to physical activity and the ability to cope with negative emotions (and, marginally, prison regime), supporting the relevance of these factors.

## Discussion

This study described the mental health profile of incarcerated adults across multiple domains. We then explored factors associated with resilience in an additional, data-informed analysis; these findings should be interpreted as hypothesis-generating.

### Mental health characterization

The present study identified that, among currently incarcerated individuals, the most frequently reported mental disorder diagnoses were schizophrenia (34.6%), personality disorders (17.3%), depression (17.3%), and substance use disorders (11.1%). Previous literature reviews published in 2022 and 2024 have similarly reported depression, stress, anxiety, substance use disorders, and personality disorders as among the most prevalent mental and behavioural conditions in prison populations [2, 7, 9]. In addition, 33.0% of participants in the present sample reported having received psychological or psychiatric care prior to incarceration. Comparable proportions have been described in other prison-based studies, including a Canadian study reporting that 60.4% of incarcerated individuals had accessed psychological or psychiatric services prior to imprisonment [46].

With respect to lifetime psychoactive substance use, tobacco and alcohol were the most frequently reported substances in the present sample, followed by cannabinoids and cocaine. Existing studies consistently document a high prevalence of substance use histories among incarcerated populations, with estimates ranging between 50% and 80% [47–49]. Research conducted in prison settings has further described alcohol, cocaine, cannabis, and non-prescription psychotropic drugs as the most commonly reported substances among single-substance users, whereas cannabis, alcohol, psychotropic drugs, cocaine, and heroin are frequently reported among individuals with patterns of poly-substance use [50, 51].

The analysis of mean scores on the DASS-21 subscales showed values of 4.7 for anxiety, 4.9 for depression, and 6.2 for stress among the incarcerated individuals included in this study. Previous research conducted among incarcerated young offenders in Portugal reported mean scores of 5.0 for anxiety, 4.5 for depression, and 8.1 for stress, while a study conducted among incarcerated individuals in Croatia reported mean scores of 5.9 for anxiety, 6.9 for depression, and 7.8 for stress [22, 42, 52].

The present study reports a mean resilience score of 29.1 among incarcerated men, as measured by the CD-RISC-10. In the absence of validated clinical cut-off points for resilience in prison populations, this value is presented descriptively and treated as a continuous measure. Following the descriptive characterisation, resilience was defined as the primary outcome of the multivariable analyses, in view of its clinical relevance and its role as a potentially modifiable psychological construct within prison mental health care [18]. Building on this descriptive characterisation, the multivariable analyses examined associations between resilience and a range of sociodemographic, clinical, and prison-context factors.

Previous prison-based research has examined resilience alongside other psychosocial resources, such as self-acceptance and perceived social support, and has reported associations between these constructs and mental health outcomes among incarcerated individuals [21]. Within this context, the present findings support the inclusion of resilience as a relevant construct within a multidimensional approach to mental health assessment in custodial settings.

Regarding hope, the mean score on the HHI-PT was 31.2 and is reported descriptively as part of the multidimensional mental health assessment. Previous prison-based research conducted in different national contexts has examined hope among incarcerated individuals and highlighted its relevance as a psychological construct within custodial settings [53, 54]. In addition, prior studies in prison populations have reported associations between hope, resilience, and other mental health indicators [23, 48].

The PWBM-S revealed a generally satisfactory level of psychological well-being among participants, with a mean score of 96.77, which is reported descriptively. Variability in psychological well-being in custodial settings has been linked in the literature to differences in prison conditions and to broader cultural and institutional contexts that shape the prison climate, the availability of social support, and processes of psychological adaptation. Within this perspective, the present findings are interpreted as contextual information contributing to a multidimensional understanding of mental health, rather than as indicators of relative levels of well-being across settings [48, 49, 54, 55].

### Factors associated with resilience

Multivariable linear regression analysis was conducted to examine factors associated with resilience among incarcerated individuals. Prior psychological or psychiatric support before incarceration was positively associated with resilience. Previous prison-based studies have highlighted the relevance of prior contact with mental health services when examining psychosocial functioning, psychological adaptation, and mental health outcomes during incarceration [56].

Prison regime was associated with resilience, with higher scores observed among individuals in less restrictive regimes. Previous prison-based studies have similarly reported positive associations between less restrictive prison regimes and resilience among incarcerated individuals [57].

The ability to cope with negative emotions was positively associated with resilience among incarcerated individuals. Previous prison-based studies have similarly reported positive associations between emotional regulation capacities and resilience, as well as other mental health indicators, including anxiety, depression, and stress [58].

Participation in physical activity was positively associated with resilience among incarcerated individuals. Previous prison-based research has similarly reported positive associations between participation in physical activity and resilience, as well as other psychological indicators, in prison populations [59].

Experiences of verbal and/or physical aggression within the prison context were associated with lower resilience scores among incarcerated individuals. Consistent with this, previous prison-based research, including systematic reviews and meta-analyses, has reported negative associations between exposure to aggressive or traumatic events and resilience in incarcerated populations [60].

Face-to-face contact with family and friends was positively associated with resilience among incarcerated individuals. Previous prison-based research has similarly reported positive associations between social contact or social support and resilience, as well as other mental health indicators, in incarcerated populations [61].

Reflecting on or revisiting the reasons for incarceration was positively associated with resilience among incarcerated individuals. Previous prison-based research has also examined reflective practices in prison settings and reported positive associations with psychosocial outcomes, including resilience [62].

Engagement in relaxation techniques has been examined in previous prison-based research and described as a relevant psychosocial factor in relation to resilience and mental health outcomes in incarcerated populations [63, 64].

Despite the inclusion of several theoretically relevant variables in Model A, some factors did not show a statistically significant association with resilience among incarcerated individuals in the present analysis. This absence of association should not be interpreted as evidence of irrelevance across incarcerated contexts.

With regard to the diagnosis of mental illness, both prior to and during incarceration, previous prison-based studies have described associations between engagement with mental health services and psychosocial factors relevant in custodial settings, including emotional support and perceived social support, which have been described as associated with resilience in incarcerated populations [65–67].

Contact by letter or telephone with family and friends: previous studies conducted in prisons have reported positive associations between these forms of indirect communication and psychosocial adjustment, including resilience, among incarcerated individuals [68–70].

With regard to the perceived adequacy of reintegration planning, previous prison-based studies have examined reintegration planning and continuity of support during the transition from custody to the community, describing associations with resilience in incarcerated populations or individuals transitioning to the community [69].

With regard to religious practices, previous studies conducted in United States prison settings have described associations between participation in religious activities and resilience among incarcerated individuals [70].

### Strengths and limitations

This study addresses the globally recognised importance of mental health in custodial settings and emphasises the urgent need for targeted public-health policies worldwide.

It represents one of the most comprehensive studies conducted in Portugal to characterise the mental health status of the prison population, covering key dimensions such as depression, anxiety, stress, resilience, hope, psychoactive substance use, and perceived psychological well-being. In addition, the study identifies factors associated with resilience.

At the same time, several limitations should be taken into account when interpreting the findings. The cross-sectional nature of the study does not allow for conclusions regarding causality or temporal sequencing, and the observed relationships should therefore be understood as associations captured at a single point in time. In addition, the use of a non-probability convenience sampling strategy, although common in prison research due to structural, security-related, and access constraints, may have introduced selection bias and limits the generalisability of the findings beyond the study sample. Participation was voluntary, and those who agreed to take part may have differed from non-participants in relevant ways, such as motivation, literacy, psychological distress, or engagement with health services. An additional limitation relates to the inability to calculate a formal response rate. Due to operational and security constraints inherent to custodial settings, recruitment was mediated by clinical services staff, and systematic records of the total number of individuals approached, eligible, or declining participation were not available across establishments. As a result, attrition bias cannot be quantified and the representativeness of the sample cannot be formally assessed.

Resilience was selected for further modelling after reviewing the descriptive profile, which may increase the risk of capitalising on chance. Consequently, these results are exploratory and require confirmation in future studies with pre-specified outcomes.

Some measurement-related limitations should also be acknowledged. The study included a self-developed sociodemographic and legal–criminal data collection tool that was not formally validated. However, it was informed by prior empirical literature and used exclusively to collect factual and contextual variables for sample characterisation, rather than to measure latent psychosocial constructs or generate composite scores. In addition, substance use was assessed using an abbreviated version of the ASSIST, which may have reduced the depth and precision of measurement compared with the full validated instrument. The adapted items were analysed individually rather than as a composite scale, and no formal psychometric validation of the abbreviated version was undertaken. The reliance on self-report measures further introduces the potential for reporting biases, including social desirability and under-reporting, which may be particularly salient in custodial settings where concerns about confidentiality, institutional mistrust, or fear of repercussions can influence how experiences and symptoms are reported.

Finally, the study sample comprised exclusively male incarcerated individuals, which limits the applicability of the findings to incarcerated women, whose mental health profiles and protective processes may differ. The research was conducted within prisons in mainland Portugal, and institutional, cultural, and policy-related characteristics specific to this context may also constrain the transferability of the findings to other correctional systems. Future research using longitudinal designs, broader and more comprehensive measurement strategies, and mixed-methods approaches would be particularly valuable in clarifying temporal pathways, addressing confounding more thoroughly, and capturing the dynamic nature of mental health and resilience in custodial settings.

### Practical implications for prison mental health care in Portugal

The findings of this study underscore the clinical relevance of resilience within the mental health profile of incarcerated adults, indicating its importance for prison mental health care planning. Resilience promotion should be integrated into routine care delivered by multidisciplinary mental health teams, through the adaptation of existing programmes or the implementation of brief, structured interventions focused on strengthening coping skills, emotional regulation, and psychological adjustment to incarceration, in a manner compatible with different prison regimes.

The effectiveness of such interventions should be systematically monitored through the use of validated assessment instruments, allowing for the evaluation of outcomes, comparison of practices across institutions, and support of evidence-based decision-making. To ensure applicability at a national level, programmes should be designed to be flexible, replicable, and accompanied by clear operational guidance, enabling their implementation by mental health teams across diverse prison settings.

In addition, the findings highlight the relevance of family contact as a factor associated with resilience, underscoring the need for discussion at a governmental level regarding the duration, frequency, and conditions of family contact in custodial settings. Such discussions should involve policy-makers, prison administration, and mental health teams, recognising family contact as a structural determinant of mental health and social reintegration. Finally, the establishment of regular forums for collaboration between prison mental health teams may facilitate the exchange of experiences and contribute to greater consistency and coherence of practices at a national level.

## Conclusions

Mean DASS-21 scores showed relatively low central tendency values, with similar averages for anxiety and depression and slightly higher mean scores for the stress subscale. The wide score range observed across all subscales (minimum 0; maximum 21) indicates substantial intra-sample heterogeneity, reflecting the coexistence of participants reporting minimal symptoms and others reporting markedly higher levels of emotional distress. Resilience (CD-RISC-10) showed an intermediate mean value, accompanied by considerable dispersion, reflecting meaningful individual variability in psychological adaptation within the prison context. Mean levels of hope (HHI-PT) and psychological well-being (PWBM-S) were located in the upper range of the possible scale scores, describing elevated levels of self-reported positive psychological functioning in the sample, despite the adverse conditions associated with imprisonment. Regarding substance use, an increased risk of dependence was observed for tobacco, alcohol, cannabinoids and cocaine.

In Model A, after simultaneous adjustment for all predefined covariates, prior psychological or psychiatric support before incarceration, placement in an open prison regime within the institution, greater self-reported ability to manage negative emotions, frequent engagement in physical activity, regular face-to-face contact with family and friends, frequent practice of relaxation techniques, and regular reflection on the reasons for incarceration were positively associated with resilience. In contrast, exposure to verbal and/or physical aggression within the prison context was inversely associated with resilience.

Multiple mental health domains were characterised; however, modelling was undertaken for a single domain selected on the basis of its clinical relevance, namely resilience.

From the robustness analysis comparing Model A and Models B, the following variables remained stable across all models: an open regime within the prison setting, regular face-to-face contact with family and friends, and frequent engagement in physical activity, all of which were associated with higher resilience. Reflecting on the reasons for incarceration also maintained a consistent association. Prior psychological support was attenuated and did not remain statistically significant in Models B after adjustment for emotional symptomatology. The ability to cope with negative emotions and the use of relaxation techniques did not constitute stable predictors. Exposure to verbal and/or physical aggression showed a negative association in some models but was not robust across all adjustments.

From the penalised regression analyses (Elastic Net/LASSO), the Elastic Net model highlighted frequent engagement in physical activity as the most prominent factor, followed by prior psychological support, the ability to cope with negative emotions, regular face-to-face contact with family and friends, a more open prison regime, and age. In the LASSO analysis, only a highly parsimonious subset of variables was retained, predominantly indicators related to physical activity and the ability to cope with negative emotions, with prison regime playing a marginal role, supporting the relevance of these factors.

The findings further contribute to raising awareness of the specific challenges faced by individuals deprived of liberty, fostering informed debate and actions aimed at safeguarding human rights and improving mental health outcomes in prison systems. Resilience is particularly salient in this context, not only as a protective factor for mental health but also due to its connection with cultural norms that value strength, self-sufficiency and emotional invulnerability. Such expectations may lead prisoners to conceal their vulnerabilities and to avoid seeking support, thereby compromising their psychological well-being. Consequently, it is recommended that resilience be examined further through longitudinal and qualitative research, thereby providing a more comprehensive understanding of its trajectory and the subjective experiences of individuals in prison. Moreover, these findings carry important implications for clinical practice, highlighting the need to develop and implement interventions aimed at strengthening resilience within this population, as a key component in promoting mental health and fostering positive adaptation to the prison environment.

Considering that psychosocial factors and mental health needs may differ substantially between sexes, future research should include representative samples of incarcerated women to better understand their specific needs and develop tailored interventions for this population.

## Supplementary Information

Below is the link to the electronic supplementary material.

Supplementary Material 1

Supplementary Material 2

Supplementary Material 3

Supplementary Material 4

Supplementary Material 5

Supplementary Material 6

Supplementary Material 7

## Data Availability

The datasets used and/or analysed during the current study are available from the corresponding author upon reasonable request.
