# Supplementary material for: Resilience and associated factors within the mental health profile of incarcerated adults in Portugal: a cross-sectional study
Source: BMC Psychiatry. 2026 Jan 29;26:203. doi: 10.1186/s12888-026-07852-1 (PMC12924268; doi:10.1186/s12888-026-07852-1)
Supplement: Supplementary file 1 — Supplementary Material 1 [file 12888_2026_7852_MOESM1_ESM.pdf]

Supplementary File 3 - Model B - Stress

Variable	Unstandardized B	p-Value	95% CI for B	Tolerance	VIF
Age	0.024	p=0.376	[-0.029; 0.078]	0.678	1.474
Has children (Yes)	0.168	p=0.799	[-1.124; 1.459]	0.713	1.403
Psychological support before incarceration (Yes)	1.321	p=0.056	[-0.034; 2.677]	0.698	1.433
History of diagnosed mental disorder prior to incarceration (yes)	1.386	p=0.242	[-0.937; 3.708]	0.439	2.276
Current diagnosed mental disorder (yes)	-0.781	p=0.490	[-3.000; 1.438]	0.476	2.099
Time incarcerated (days)	-1.216E-5	p=0.970	[-0.001; 0.001]	0.849	1.178
Prison regime					
The common prison regime	Ref				
The security regime	1.683	p=0.125	[-0.466; 3.833]	0.803	1.245
**The open regime within prison**	**2.730**	**p=0.004**	**[0.865; 4.594]**	**0.826**	**1.211**
The open regime outside prison	1.896	p=0.332	[-1.939; 5.731]	0.874	1.144
Stimulating activities					
I completely disagree	Ref				
I disagree	-0.093	p=0.929	[-2.152; 1.965]	0.478	2.094
I neither agree nor disagree	-0.576	p=0.577	[-2.604; 1.451]	0.473	2.113
I agree	-0.681	p=0.484	[-2.592; 1.230]	0.369	2.707
I completely agree	-0.470	p=0.668	[-2.618; 1.679]	0.488	2.048
The ability to cope with negative emotions					
I completely disagree	Ref				
I disagree	-1.629	p=0.265	[-4.499; 1.241]	0.369	2.712
I neither agree nor disagree	-0.576	p=0.667	[-3.209; 2.057]	0.287	3.480
I agree	1.127	p=0.370	[-1.340; 3.595]	0.187	5.360
**I completely agree**	**2.986**	**p=0.029**	**[0.303; 5.670]**	**0.279**	**3.588**

There is adequate planning for reintegration					
I completely disagree	Ref				
I disagree	-1.621	p=0.096	[-3.528; 0.286]	0.467	2.141
I neither agree nor disagree	-1.103	p=0.302	[-3.203; 0.996]	0.538	1.860
I agree	-0.111	p=0.906	[-1.948; 1.726]	0.413	2.424
I completely agree	-0.813	p=0.460	[-2.975; 1.349]	0.487	2.054
There is prejudice due to having been incarcerated					
I completely disagree	Ref				
I disagree	0.106	p=0.931	[-2.310; 2.522]	0.379	2.640
I neither agree nor disagree	-0.582	p=0.635	[-2.988; 1.824]	0.321	3.111
I agree	0.125	p=0.913	[-2.118; 2.367]	0.248	4.031
I completely agree	0.403	p=0.722	[-1.823; 2.628]	0.331	3.020
Face-to-face contact with family and friends					
Never	Ref				
Once a month	1.577	p=0.083	[-0.204; 3.359]	0.495	2.019
**Once every two weeks**	**3.463**	**p=0.003**	**[1.168; 5.759]**	**0.613**	**1.631**
**Once a week**	**3.046**	**p=0.002**	**[1.129; 4.963]**	**0.480**	**2.085**
**Twice or more times a week**	**2.426**	**p=0.011**	**[0.556; 4.296]**	**0.395**	**2.531**
Contact by letter or telephone with friends or family					
Never	Ref				
Once every two weeks	2.201	p=0.103	[-0.450; 4.851]	0.500	2.000
Once a week	-0.819	p=0.541	[-3.450; 1.812]	0.482	2.074
Twice a week	-1.217	p=0.415	[-4.144; 1.710]	0.537	1.863
More than twice a week	-0.011	p=0.992	[-2.051; 2.029]	0.303	3.296
Physical activity					
Never	Ref				

Once a week	1.500	p=0.095	[-0.260; 3.261]	0.670	1.493
Twice a week	0.290	p=0.775	[-1.698; 2.278]	0.672	1.489
Three times a week	1.820	p=0.085	[-0.249; 3.890]	0.670	1.492
**Four or more times a week**	**2.932**	**p<0.001**	**[1.291; 4.574]**	**0.469**	**2.133**
Practice of relaxation techniques					
Never	Ref				
Once a week	-1.035	p=0.205	[-2.636; 0.567]	0.831	1.204
Twice a week	1.091	p=0.398	[-1.443; 3.625]	0.867	1.153
Three times a week	-0.017	p=0.992	[-3.309; 3.276]	0.864	1.158
Four or more times a week	2.037	p=0.083	[-0.266; 4.340]	0.808	1.237
Experiences of verbal and/or physical aggression					
Never	Ref				
Once a month	-1.464	p=0.087	[-3.138; 0.211]	0.796	1.256
Twice a month	-0.550	p=0.629	[-2.782; 1.682]	0.841	1.188
Three times a month	0.519	p=0.711	[-2.227; 3.266]	0.872	1.147
Four or more times a month	-0.706	p=0.506	[-2.790; 1.377]	0.795	1.258
Religious practices					
Never	Ref				
Once every two weeks	0.184	p=0.843	[-1.639; 2.007]	0.799	1.252
Once a week	1.019	p=0.178	[-0.465; 2.502]	0.745	1.342
Twice a week	-1.267	p=0.375	[-4.074; 1.539]	0.835	1.198
More than twice a week	1.225	p=0.153	[-0.456; 2.907]	0.741	1.350
Reflect on or revisit the reasons for their incarceration					
Never	Ref				
Once every two weeks	2.562	p=0.081	[-0.317; 5.441]	0.555	1.802
**Once a week**	**3.812**	**p=0.008**	**[0.980; 6.645]**	**0.534**	**1.871**
Twice a week	-0.949	p=0.548	[-4.053; 2.156]	0.596	1.678

More than twice a week	1.281	p=0.219	[-0.762; 3.323]	0.341	2.930
**Stress**	**-0.286**	**p<0.001**	**[-0.408; -0.164]**	**0.715**	**1.398**
