# Supplementary material for: Resilience and associated factors within the mental health profile of incarcerated adults in Portugal: a cross-sectional study
Source: BMC Psychiatry. 2026 Jan 29;26:203. doi: 10.1186/s12888-026-07852-1 (PMC12924268; doi:10.1186/s12888-026-07852-1)
Supplement: Supplementary file 2 — Supplementary Material 2 [file 12888_2026_7852_MOESM2_ESM.pdf]

Supplementary File 4 - Model B - Anxiety

Variable	Unstandardized B	P-Value	95% CI for B	Tolerance	VIF
Age	0.029	p=0.280	[-0.024; 0.081]	0.685	1.460
Has children (Yes)	0.293	p=0.650	[-0.977; 1.563]	0.712	1.405
Psychological support before incarceration (Yes)	1.220	p=0.073	[-0.112; 2.552]	0.698	1.433
History of diagnosed mental disorder prior to incarceration (yes)	1.669	p=0.152	[-0.616; 3.954]	0.438	2.283
Current diagnosed mental disorder (yes)	-1.314	p=0.240	[-3.507; 0.879]	0.471	2.124
Time incarcerated (days)	5.251E-5	p=0.870	[-0.001; 0.001]	0.847	1.180
Prison regime					
The common prison regime	Ref				
The security regime	1.763	p=0.101	[-0.348; 3.873]	0.804	1.243
**The open regime within prison**	**2.649**	**p=0.005**	**[0.820; 4.479]**	**0.828**	**1.208**
The open regime outside prison	1.661	p=0.386	[-2.101; 5.422]	0.877	1.141
Stimulating activities					
I completely disagree	Ref				
I disagree	-0.204	p=0.843	[-2.227; 1.819]	0.477	2.095
I neither agree nor disagree	-0.574	p=0.571	[-2.566; 1.417]	0.473	2.113
I agree	-0.647	p=0.498	[-2.524; 1.229]	0.370	2.706
I completely agree	-0.502	p=0.640	[-2.612; 1.608]	0.489	2.047
The ability to cope with negative emotions					
I completely disagree	Ref				
I disagree	-2.003	p=0.164	[-4.826; 0.820]	0.368	2.718
I neither agree nor disagree	-1.141	p=0.389	[-3.740; 1.457]	0.285	3.513
I agree	0.283	p=0.821	[-2.171; 2.737]	0.182	5.494
I completely agree	2.407	p=0.074	[-0.238; 5.051]	0.277	3.609

There is adequate planning for reintegration					
I completely disagree	Ref				
I disagree	-1.727	p=0.071	[-3.601; 0.147]	0.467	2.143
I neither agree nor disagree	-1.055	p=0.316	[-3.117; 1.008]	0.538	1.859
I agree	-0.267	p=0.771	[-2.073; 1.539]	0.412	2.427
I completely agree	-0.688	p=0.525	[-2.812; 1.436]	0.487	2.054
There is prejudice due to having been incarcerated					
I completely disagree	Ref				
I disagree	0.201	p=0.868	[-2.172; 2.574]	0.379	2.638
I neither agree nor disagree	-0.311	p=0.796	[-2.677; 2.055]	0.321	3.118
I agree	0.285	p=0.799	[-1.917; 2.488]	0.248	4.028
I completely agree	0.367	p=0.741	[-1.814; 2.548]	0.333	3.005
Face-to-face contact with family and friends					
Never	Ref				
Once a month	1.519	p=0.088	[-0.228; 3.267]	0.497	2.012
**Once every two weeks**	**3.323**	**p=0.004**	**[1.069; 5.578]**	**0.613**	**1.630**
**Once a week**	**2.993**	**p=0.002**	**[1.113; 4.874]**	**0.481**	**2.078**
**Twice or more times a week**	**2.485**	**p=0.008**	**[0.649; 4.321]**	**0.396**	**2.527**
Contact by letter or telephone with friends or family					
Never	Ref				
Once every two weeks	2.295	p=0.084	[-0.308; 4.897]	0.500	1.998
Once a week	-0.668	p=0.612	[-3.253; 1.917]	0.482	2.075
Twice a week	-1.031	p=0.482	[-3.908; 1.846]	0.537	1.863
More than twice a week	0.037	p=0.971	[-1.967; 2.041]	0.303	3.296
Physical activity					
Never	Ref				

Once a week	1.534	p=0.082	[-0.195; 3.263]	0.670	1.493
Twice a week	0.369	p=0.711	[-1.584; 2.322]	0.672	1.488
Three times a week	1.815	p=0.080	[-0.218; 3.848]	0.670	1.492
**Four or more times a week**	**2.775**	**p<.001**	**[1.163; 4.387]**	**0.469**	**2.132**
Practice of relaxation techniques					
Never	Ref				
Once a week	-1.020	p=0.203	[-2.593; 0.552]	0.832	1.202
Twice a week	1.252	p=0.324	[-1.237; 3.742]	0.867	1.153
Three times a week	0.555	p=0.736	[-2.683; 3.793]	0.862	1.160
Four or more times a week	1.854	p=0.108	[-0.410; 4.117]	0.808	1.238
Experiences of verbal and/or physical aggression					
Never	Ref				
**Once a month**	**-1.839**	**p=0.028**	**[-3.478; -0.199]**	**0.801**	**1.248**
Twice a month	-0.401	p=0.720	[-2.594; 1.792]	0.842	1.188
Three times a month	0.379	p=0.782	[-2.317; 3.075]	0.874	1.145
Four or more times a month	-0.513	p=0.623	[-2.557; 1.532]	0.797	1.254
Religious practices					
Never	Ref				
Once every two weeks	0.067	p=0.941	[-1.724; 1.858]	0.799	1.252
Once a week	1.041	p=0.161	[-0.416; 2.498]	0.745	1.341
Twice a week	-0.763	p=0.588	[-3.529; 2.004]	0.830	1.206
More than twice a week	1.108	p=0.187	[-0.541; 2.756]	0.744	1.345
Reflect on or revisit the reasons for their incarceration					
Never	Ref				
Once every two weeks	2.515	p=0.081	[-0.313; 5.343]	0.555	1.801
**Once a week**	**3.570**	**p=0.012**	**[0.789; 6.351]**	**0.535**	**1.869**
Twice a week	-0.729	p=0.639	[-3.780; 2.323]	0.595	1.680

More than twice a week	1.254	p=0.220	[-0.750; 3.257]	0.342	2.921
**Anxiety**	**-0.402**	**p<0.001**	**[-0.526; -0.278]**	**0.715**	**1.400**
