# Supplementary material for: Resilience and associated factors within the mental health profile of incarcerated adults in Portugal: a cross-sectional study
Source: BMC Psychiatry. 2026 Jan 29;26:203. doi: 10.1186/s12888-026-07852-1 (PMC12924268; doi:10.1186/s12888-026-07852-1)
Supplement: Supplementary file 3 — Supplementary Material 3 [file 12888_2026_7852_MOESM3_ESM.pdf]

Supplementary File 5 - Model B - Depression

Variable	Unstandardized B	P-Value	95% CI for B	Tolerance	VIF
Age	0.025	p=0.351	[-0.028; 0.078]	0.682	1.466
Has children (Yes)	0.254	p=0.697	[-1.024; 1.532]	0.712	1.404
Psychological support before incarceration (Yes)	1.154	p=0.092	[-0.190; 2.498]	0.694	1.441
History of diagnosed mental disorder prior to incarceration (yes)	1.480	p=0.206	[-0.817; 3.777]	0.439	2.277
Current diagnosed mental disorder (yes)	-0.466	p=0.674	[-2.641; 1.710]	0.485	2.063
Time incarcerated (days)	-5.709E-5	p=0.860	[-0.001; 0.001]	0.849	1.178
Prison regime					
The common prison regime	Ref				
The security regime	1.534	p=0.157	[-0.594; 3.661]	0.802	1.247
**The open regime within prison**	**2.385**	**p=0.011**	**[0.544; 4.227]**	**0.828**	**1.208**
The open regime outside prison	1.512	p=0.433	[-2.274; 5.297]	0.877	1.140
Stimulating activities					
I completely disagree	Ref				
I disagree	-0.136	p=0.896	[-2.172; 1.900]	0.478	2.094
I neither agree nor disagree	-0.788	p=0.441	[-2.794; 1.219]	0.473	2.115
I agree	-0.875	p=0.364	[-2.768; 1.017]	0.368	2.715
I completely agree	-0.615	p=0.570	[-2.739; 1.509]	0.489	2.047
The ability to cope with negative emotions					
I completely disagree	Ref				
I disagree	-1.960	p=0.176	[-4.802; 0.882]	0.368	2.718
I neither agree nor disagree	-0.904	p=0.497	[-3.513; 1.706]	0.286	3.496
I agree	0.645	p=0.606	[-1.810; 3.100]	0.184	5.428

I completely agree	2.361	p=0.084	[-0.317; 5.039]	0.274	3.653
There is adequate planning for reintegration					
I completely disagree	Ref				
I disagree	-1.524	p=0.113	[-3.409; 0.360]	0.468	2.139
I neither agree nor disagree	-0.975	p=0.357	[-3.051; 1.101]	0.538	1.860
I agree	0.127	p=0.891	[-1.690; 1.944]	0.412	2.426
I completely agree	-0.519	p=0.634	[-2.658; 1.620]	0.486	2.056
There is prejudice due to having been incarcerated					
I completely disagree	Ref				
I disagree	-0.171	p=0.888	[-2.555; 2.213]	0.380	2.629
I neither agree nor disagree	-0.885	p=0.463	[-3.256; 1.485]	0.324	3.087
I agree	-0.065	p=0.954	[-2.275; 2.144]	0.250	4.001
I completely agree	0.125	p=0.911	[-2.067; 2.317]	0.334	2.995
Face-to-face contact with family and friends					
Never	Ref				
Once a month	1.504	p=0.094	[-0.256; 3.265]	0.496	2.017
**Once every two weeks**	**3.204**	**p=0.006**	**[0.929; 5.478]**	**0.610**	**1.638**
**Once a week**	**2.940**	**p=0.002**	**[1.045; 4.836]**	**0.480**	**2.085**
**Twice or more times a week**	**2.372**	**p=0.012**	**[0.523; 4.221]**	**0.395**	**2.531**
Contact by letter or telephone with friends or family					
Never	Ref				
Once every two weeks	2.452	p=0.067	[-0.173; 5.077]	0.498	2.006
Once a week	-0.587	p=0.658	[-3.190; 2.015]	0.482	2.076
Twice a week	-0.687	p=0.642	[-3.588; 2.214]	0.535	1.871
More than twice a week	-0.025	p=0.981	[-2.042; 1.993]	0.303	3.295
Physical activity					

Never	Ref				
Once a week	1.483	p=0.095	[-0.258; 3.223]	0.670	1.492
Twice a week	-0.034	p=0.973	[-1.997; 1.928]	0.674	1.483
Three times a week	1.517	p=0.146	[-0.532; 3.566]	0.669	1.496
**Four or more times a week**	**2.556**	**p=0.002**	**[0.930; 4.181]**	**0.467**	**2.140**
Practice of relaxation techniques					
Never	Ref				
Once a week	-0.989	p=0.220	[-2.572; 0.593]	0.832	1.202
Twice a week	1.324	p=0.300	[-1.182; 3.830]	0.867	1.154
Three times a week	0.341	p=0.837	[-2.916; 3.598]	0.863	1.159
**Four or more times a week**	**2.311**	**p=0.046**	**[0.040; 4.582]**	**0.813**	**1.230**
Experiences of verbal and/or physical aggression					
Never	Ref				
**Once a month**	**-1.810**	**p=0.032**	**[-3.461; -0.160]**	**0.801**	**1.248**
Twice a month	-0.722	p=0.520	[-2.923; 1.479]	0.846	1.182
Three times a month	0.253	p=0.855	[-2.460; 2.966]	0.874	1.144
Four or more times a month	-0.544	p=0.604	[-2.604; 1.517]	0.795	1.257
Religious practices					
Never	Ref				
Once every two weeks	0.094	p=0.918	[-1.709; 1.898]	0.799	1.252
Once a week	1.009	p=0.177	[-0.458; 2.477]	0.745	1.342
Twice a week	-1.111	p=0.432	[-3.888; 1.666]	0.834	1.199
More than twice a week	0.908	p=0.283	[-0.751; 2.566]	0.744	1.344
Reflect on or revisit the reasons for their incarceration					
Never	Ref				
Once every two weeks	2.420	p=0.096	[-0.428; 5.268]	0.555	1.803
**Once a week**	**3.508**	**p=0.014**	**[0.709; 6.308]**	**0.535**	**1.869**

Twice a week	-0.996	p=0.524	[-4.065; 2.074]	0.596	1.678
More than twice a week	1.233	p=0.231	[-0.784; 3.250]	0.342	2.921
**Depression**	**-0.364**	**p<0.001**	**[-0.488; -0.241]**	**0.730**	**1.370**
