# Supplementary material for: Resilience and associated factors within the mental health profile of incarcerated adults in Portugal: a cross-sectional study
Source: BMC Psychiatry. 2026 Jan 29;26:203. doi: 10.1186/s12888-026-07852-1 (PMC12924268; doi:10.1186/s12888-026-07852-1)
Supplement: Supplementary file 4 — Supplementary Material 4 [file 12888_2026_7852_MOESM4_ESM.pdf]

UNIVERSIDADE DE ÉVORA CHRC COMPREHENSIVE HEALTH RESEARCH CENTRE NOVA UNIVERSIDADE NOVA DE LISBOA

SOCIODEMOGRAPHIC AND LEGAL-CRIMINAL QUESTIONNAIRE

1) Age: ___________

2) Sex: Female [ ]   Male [ ]

3) Do you have children?? Yes [ ]   No [ ]

Did you receive psychiatric/psychological care prior to detention?

Yes [ ] No [ ]   If yes, what was the reason?:___________________

Have you ever been diagnosed with a mental disorder prior to detention?

Yes [ ] No [ ]   If yes, which one?______________________

Do you currently have a diagnosed mental disorder?

Yes [ ] No [ ]   If yes, which one?______________________

4) Length of incarceration _____ Years _____ Months _____ Weeks _____ Days _____

5) Under which prison regime are you currently serving your sentence?

The common prison regime	The security regime	The open regime within prison	The open regime outside prison

Please select the option that best applies to you by circling the one you wish to choose.

6) Do you agree that the prison provides stimulating and diverse activities for your personal, emotional, and professional development?

I completely disagree	I disagree	I neither agree nor disagree	I agree	I completely agree

7) Do you consider yourself able to cope with negative emotions (such as sadness, anger, fear, distress) arising from your imprisonment?

I completely disagree	I disagree	I neither agree nor disagree	I agree	I completely agree

8) Do you consider that there is adequate planning tailored to your needs to support your reintegration into society after serving your sentence?

I completely disagree	I disagree	I neither agree nor disagree	I agree	I completely agree

9) Do you consider that there is negative prejudice associated with being incarcerated, and that this may hinder your reintegration into society?

I completely disagree	I disagree	I neither agree nor disagree	I agree	I completely agree

Considering the past month, please answer the following questions:

10) On average, how often have you had face-to-face contact with your friends or family?

Never	Once a month	Once every two weeks	Once a week	Twice or more times a week

11) On average, how often have you had contact by letter/telephone with your friends or family?

Never	Once every two weeks	Once a week	Twice a week	Twice or more times a week

12) On average, how often have you engaged in physical exercise?

Never	Once a week	Twice a week	Three times a week	Four or more times a week

13) On average, how often have you practised any relaxation technique, meditation, breathing control, mindfulness, or muscle relaxation?

Never	Once a week	Twice a week	Three times a week	Four or more times a week

14) On average, how often have you been verbally and/or physically assaulted?

Never	Once a month	Twice a month	Three times a month	Four or more times a month

15) On average, how often have you engaged in religious practices, such as praying, attending mass, listening to mass on the radio or television, or participating in support groups (e.g., Samaritans)?

Never	Once every two weeks	Once a week	Twice a week	More than twice a week

16) On average, how often have you relived or thought about the reason for your imprisonment??

Never	Once every two weeks	Once a week	Twice a week	More than twice a week
