# Supplementary material for: Resilience and associated factors within the mental health profile of incarcerated adults in Portugal: a cross-sectional study
Source: BMC Psychiatry. 2026 Jan 29;26:203. doi: 10.1186/s12888-026-07852-1 (PMC12924268; doi:10.1186/s12888-026-07852-1)
Supplement: Supplementary file 5 — Supplementary Material 5 [file 12888_2026_7852_MOESM5_ESM.pdf]

Supplementary File 2 - Model B - DASS 21

Variable	Unstandardized B	P-Value	95% CI for B	Tolerance	VIF
Age	0.028	p=0.296	[-0.025; 0.081]	0.676	1.479
Has children (Yes)	0.326	p=0.614	[-0.942; 1.594]	0.711	1.407
Psychological support before incarceration (Yes)	1.140	p=0.093	[-0.193; 2.474]	0.694	1.441
History of diagnosed mental disorder prior to incarceration (yes)	1.682	p=0.148	[-0.599; 3.964]	0.438	2.283
Current diagnosed mental disorder (yes)	-1.098	p=0.327	[-3.298; 1.101]	0.466	2.145
Time incarcerated (days)	2.059E-5	p=0.949	[-0.001; 0.001]	0.844	1.184
Prison regime					
The common prison regime	Ref				
The security regime	1.665	p=0.122	[-0.447; 3.777]	0.800	1.250
**The open regime within prison**	**2.496**	**p=0.008**	**[0.662; 4.331]**	**0.821**	**1.219**
The open regime outside prison	1.524	p=0.427	[-2.240; 5.288]	0.872	1.146
Stimulating activities					
I completely disagree	Ref				
I disagree	-0.209	p=0.839	[-2.229; 1.810]	0.477	2.095
I neither agree nor disagree	-0.681	p=0.502	[-2.673; 1.310]	0.472	2.119
I agree	-0.757	p=0.429	[-2.635; 1.122]	0.368	2.719
I completely agree	-0.567	p=0.598	[-2.674; 1.541]	0.488	2.050
The ability to cope with negative emotions					
I completely disagree	Ref				
I disagree	-2.114	p=0.142	[-4.936; 0.708]	0.367	2.726
I neither agree nor disagree	-1.250	p=0.345	[-3.847; 1.347]	0.284	3.520
I agree	0.134	p=0.914	[-2.320; 2.589]	0.181	5.514

I completely agree	2.135	p=0.116	[-0.526; 4.796]	0.273	3.667
There is adequate planning for reintegration					
I completely disagree	Ref				
I disagree	-1.667	p=0.081	[-3.539; 0.204]	0.466	2.145
I neither agree nor disagree	-0.998	p=0.341	[-3.058; 1.061]	0.537	1.861
I agree	-0.124	p=0.893	[-1.933; 1.685]	0.409	2.445
I completely agree	-0.561	p=0.604	[-2.685; 1.563]	0.485	2.062
There is prejudice due to having been incarcerated					
I completely disagree	Ref				
I disagree	0.059	p=0.961	[-2.314; 2.432]	0.378	2.649
I neither agree nor disagree	-0.489	p=0.686	[-2.858; 1.880]	0.319	3.136
I agree	0.183	p=0.871	[-2.020; 2.385]	0.247	4.041
I completely agree	0.250	p=0.822	[-1.935; 2.435]	0.330	3.026
Face-to-face contact with family and friends					
Never	Ref				
Once a month	1.475	p=0.098	[-0.273; 3.224]	0.495	2.022
**Once every two weeks**	**3.197**	**p=0.006**	**[0.941; 5.453]**	**0.610**	**1.639**
**Once a week**	**2.932**	**p=0.002**	**[1.051; 4.814]**	**0.479**	**2.088**
**Twice or more times a week**	**2.442**	**p=0.009**	**[0.607; 4.277]**	**0.395**	**2.534**
Contact by letter or telephone with friends or family					
Never	Ref				
Once every two weeks	2.423	p=0.068	[-0.181; 5.027]	0.498	2.007
Once a week	-0.574	p=0.663	[-3.156; 2.009]	0.481	2.078
Twice a week	-0.791	p=0.590	[-3.672; 2.091]	0.533	1.877
More than twice a week	0.024	p=0.981	[-1.976; 2.025]	0.303	3.296
Physical activity					

Never	Ref				
Once a week	1.523	p=0.084	[-0.204; 3.249]	0.670	1.493
Twice a week	0.203	p=0.839	[-1.754; 2.159]	0.667	1.499
Three times a week	1.663	p=0.109	[-0.373; 3.698]	0.666	1.501
**Four or more times a week**	**2.612**	**p=0.002**	**[0.993; 4.230]**	**0.464**	**2.157**
Practice of relaxation techniques					
Never	Ref				
Once a week	-1.007	p=0.209	[-2.577; 0.564]	0.831	1.204
Twice a week	1.342	p=0.290	[-1.146; 3.830]	0.865	1.156
Three times a week	0.643	p=0.697	[-2.599; 3.884]	0.857	1.166
Four or more times a week	2.006	p=0.082	[-0.259; 4.270]	0.804	1.244
Experiences of verbal and/or physical aggression					
Never	Ref				
**Once a month**	**-1.943**	**p=0.021**	**[-3.597; -0.289]**	**0.784**	**1.275**
Twice a month	-0.495	p=0.658	[-2.687; 1.697]	0.840	1.191
Three times a month	0.277	p=0.840	[-2.418; 2.973]	0.870	1.149
Four or more times a month	-0.435	p=0.677	[-2.482; 1.613]	0.792	1.263
Religious practices					
Never	Ref				
Once every two weeks	0.042	p=0.963	[-1.747; 1.831]	0.798	1.253
Once a week	1.030	p=0.165	[-0.425; 2.485]	0.745	1.342
Twice a week	-0.749	p=0.594	[-3.512; 2.013]	0.829	1.206
More than twice a week	0.975	p=0.247	[-0.679; 2.630]	0.736	1.359
Reflect on or revisit the reasons for their incarceration					
Never	Ref				
Once every two weeks	2.441	p=0.090	[-0.383; 5.266]	0.555	1.803
**Once a week**	**3.462**	**p=0.015**	**[0.681; 6.244]**	**0.533**	**1.876**

Twice a week	- 0.784	p=0.613	[-3.831; 2.263]	0.595	1.681
More than twice a week	1.247	p=0.222	[-0.757; 3.250]	0.341	2.930
**Anxiety**	**-0.338**	**p=0.002**	**[-0.550; -0.127]**	**0.245**	**4.083**
Depression.	-0.185	p=0.053	[-0.372; 0.002]	0.315	3.173
Stress	0.089	p=0.371	[-0.107; 0.285]	0.267	3.740
