# Supplementary material for: Resilience and associated factors within the mental health profile of incarcerated adults in Portugal: a cross-sectional study
Source: BMC Psychiatry. 2026 Jan 29;26:203. doi: 10.1186/s12888-026-07852-1 (PMC12924268; doi:10.1186/s12888-026-07852-1)
Supplement: Supplementary file 7 — Supplementary Material 7 [file 12888_2026_7852_MOESM7_ESM.pdf]

Supplementary file 7 - Lasso

Variable	Standardised coefficients (|β|)
[Has children =0]	0.000
[Has children =1]	0.000
[Psychological support before incarceration =0]	-0.014
[Psychological support before incarceration =1]	0.000
[History of diagnosed mental disorder prior to incarceration =0]	0.000
[History of diagnosed mental disorder prior to incarceration =1]	0.000
[Current diagnosed mental disorder =0]	0.000
[Current diagnosed mental disorder =1]	0.000
Prison regime	
The common prison regime	Ref
[The security regime =0]	0.000
[The security regime =1]	0.000
[The open regime within prison =0]	-0.025
[The open regime within prison =1]	0.000
[The open regime outside prison =0]	0.000
[The open regime outside prison =1]	0.000
Stimulating activities	
I completely disagree	Ref
[I disagree =0]	0.000
[I disagree =1]	0.000
[I neither agree nor disagree =0]	0.000
[I neither agree nor disagree =1]	0.000
[I agree =0]	0.000
[I agree =1]	0.000

[I completely agree =0]	0.000
[I completely agree =1]	0.000
The ability to cope with negative emotions	
[I completely disagree]	Ref
[I disagree =0]	0.348
[I disagree =1]	0.000
[I neither agree nor disagree =0]	0.000
[I neither agree nor disagree =1]	0.000
[I agree =0]	0.000
[I agree =1]	0.000
[I completely agree =0]	-0.423
[I completely agree =1]	0.000
There is adequate planning for reintegration	
[I completely disagree]	Ref
[I disagree =0]	0.000
[I disagree =1]	0.000
[I neither agree nor disagree =0]	0.000
[I neither agree nor disagree =1]	0.000
[I agree =0]	0.000
[I agree =1]	0.000
[I completely agree =0]	0.000
[I completely agree =1]	0.000
There is prejudice due to having been incarcerated	
[I completely disagree]	Ref
[I disagree =0]	0.000
[I disagree =1]	0.000
[I neither agree nor disagree =0]	0.000
[I neither agree nor disagree =1]	0.000
[I agree =0]	0.000

[I agree =1]	0.000
[I completely agree =0]	0.000
[I completely agree =1]	0.000
Face-to-face contact with family and friends	
[Never]	Ref
[Once a month =0]	0.000
[Once a month =1]	0.000
[Once every two weeks =0]	0.000
[Once every two weeks =1]	0.000
[Once a week =0]	0.000
[Once a week =1]	0.000
[Twice or more times a week =0]	0.000
[Twice or more times a week =1]	0.000
Contact by letter or telephone with friends or family	
[Never]	Ref
[Once every two weeks =0]	0.000
[Once every two weeks =1]	0.000
[Once a week =0]	0.000
[Once a week =1]	0.000
[Twice a week =0]	0.000
[Twice a week =1]	0.000
[More than twice a week =0]	0.000
[More than twice a week =1]	0.000
Physical activity	
[Never]	Ref
[Once a week =0]	0.000
[Once a week =1]	0.000
[Twice a week =0]	0.000
[Twice a week =1]	0.000

[Three times a week =0]	0.000
[Three times a week =1]	0.000
[Four or more times a week =0]	-0.863
[Four or more times a week =1]	0.000
Practice of relaxation techniques	
[Never]	Ref
[Once a week =0]	0.000
[Once a week =1]	0.000
[Twice a week =0]	0.000
[Twice a week =1]	0.000
[Three times a week =0]	0.000
[Three times a week =1]	0.000
[Four or more times a week =0]	0.000
[Four or more times a week =1]	0.000
Experiences of verbal and/or physical aggression	
[Never]	Ref
[Once a month =0]	0.000
[Once a month =1]	0.000
[Twice a month =0]	0.000
[Twice a month =1]	0.000
[Three times a month =0]	0.000
[Three times a month =1]	0.000
[Four or more times a month =0]	0.000
[Four or more times a month =1]	0.000
Religious practices	
[Never]	Ref
[Once every two weeks =0]	0.000
[Once every two weeks =1]	0.000
[Once a week =0]	0.000

[Once a week =1]	0.000
[Twice a week =0]	0.000
[Twice a week =1]	0.000
[More than twice a week =0]	0.000
[More than twice a week =1]	0.000
Reflect on or revisit the reasons for their incarceration	
[Never]	Ref
[Once every two weeks =0]	
[Once every two weeks =1]	0.000
[Once a week =0]	0.000
[Once a week =1]	0.000
[Twice a week =0]	0.000
[Twice a week =1]	0.000
[More than twice a week =0]	0.000
[More than twice a week =1]	0.000
[Once every two weeks =0]	0.000
Age	0.000
Time incarcerated (days)	0.000

Note: *Coefficients correspond to the standardised coefficients from the SPSS penalised regression output; interpretation focuses on factor retention.*
